# Acute perioperative-stress-induced increase of atherosclerotic plaque volume and vulnerability to rupture in apolipoprotein-E-deficient mice is amenable to statin treatment and IL-6 inhibition

**DOI:** 10.1242/dmm.018713

**Published:** 2015-09-01

**Authors:** Henrike Janssen, Christian S. Wagner, Philipp Demmer, Simone Callies, Gesine Sölter, Houra Loghmani-khouzani, Niandan Hu, Harald Schuett, Uwe J. F. Tietge, Gregor Warnecke, Jan Larmann, Gregor Theilmeier

**Affiliations:** 1 Department of Anesthesiology and Intensive Care Medicine, Hannover Medical School, 30625 Hannover, Germany; 2 Department of Anesthesiology, University of Heidelberg, 69120 Heidelberg, Germany; 3 Department of Cardiology, University Hospital Marburg, 35043 Marburg, Germany; 4 Department of Pediatrics, University of Groningen, UMCG, NL-9700 Groningen, The Netherlands; 5 Department of Cardiothoracic, Transplant and Vascular Surgery, Hannover Medical School, 30625 Hannover, GermanyGerman Centre for Lung Research (DZL), 30625 Hannover, Germany; 6 Faculty VI - Medicine and Health Sciences, Dept of Health Services Sciences, University of Oldenburg, 26129Germany

**Keywords:** Atherosclerosis, Perioperative stress, Mouse model

## Abstract

Myocardial infarction and stroke are frequent after surgical procedures and consume a considerable amount of benefit of surgical therapy. Perioperative stress, induced by surgery, is composed of hemodynamic and inflammatory reactions. The effects of perioperative stress on atherosclerotic plaques are ill-defined. Murine models to investigate the influence of perioperative stress on plaque stability and rupture are not available. We developed a model to investigate the influence of perioperative stress on plaque growth and stability by exposing apolipoprotein-E-deficient mice, fed a high cholesterol diet for 7 weeks, to a double hit consisting of 30 min of laparotomy combined with a substantial blood loss (approximately 20% of total blood volume; 400 µl). The innominate artery was harvested 72 h after the intervention. Control groups were sham and baseline controls. Interleukin-6 (IL-6) and serum amyloid A (SAA) plasma levels were determined. Plaque load, vascular smooth muscle cell (VSMC) and macrophage content were quantified. Plaque stability was assessed using the Stary score and frequency of signs of plaque rupture were assessed. High-dose atorvastatin (80 mg/kg body weight/day) was administered for 6 days starting 3 days prior to the double hit. A single dose of an IL-6-neutralizing antibody or the fusion protein gp130-Fc selectively targeting IL-6 trans-signaling was subcutaneously injected. IL-6 plasma levels increased, peaking at 6 h after the intervention. SAA levels peaked at 24 h (*n*=4, *P*<0.01). Plaque volume increased significantly with the double hit compared to sham (*n*=8, *P*<0.01). More plaques were scored as complex or bearing signs of rupture after the double hit compared to sham (*n*=5-8, *P*<0.05). Relative VSMC and macrophage content remained unchanged. IL-6-inhibition or atorvastatin, but not blocking of IL-6 trans-signaling, significantly decreased plaque volume and complexity (*n*=8, *P*<0.01). Using this model, researchers will be able to further investigate the pathophysiology of perioperative plaque stability, which can result in myocardial infarction, and, additionally, to test potential protective strategies.

## Abstract (precis)

**Summary:** We developed a model to study the dynamics of atherosclerotic plaque growth and stability following surgery, and show that IL-6 inhibition and statins beneficially affect plaque volume and complexity.

## INTRODUCTION

Perioperative stress frequently precipitates myocardial infarction. Half of the estimated 40,000 infarctions precipitated by surgery per year in Europe originate from rupture of unstable atherosclerotic plaques, and half are due to an oxygen supply-demand mismatch (Dawood et al., 1996; Cohen and Aretz, 1999; Kertai et al., 2003; Priebe, 2011; Gualandro et al., 2012; Hanson et al., 2013). It is thought that perioperative stress in response to increased blood flow demand increases heart rate and thereby imposes additional shear stress on the atherosclerotic plaque (Priebe, 2011). Perioperative stress consists of the surgical trauma and the response of the organism to this trauma (Larmann and Theilmeier, 2004). The response to trauma encompasses inflammatory and hemodynamic changes. Hemodynamic strain is thought to cause rupture of unstable plaques or blood flow reduction by pre-existing obstructive plaques, which will both result in myocardial infarction (Landesberg et al., 2009; Priebe, 2011). The incidence of myocardial infarction is increased in patients experiencing perioperative hemorrhage (Kamel et al., 2012). A number of recent studies have suggested that atherosclerotic plaques can be subject to very fast changes in volume, composition and phenotype (Shah et al., 2001; Llodra et al., 2004; Feig et al., 2009; Larmann et al., 2013). Whether plaque growth or stability, and thereby vulnerability to rupture, can be subject to change due to the inflammatory component of perioperative stress is, however, unknown.

Our group and others have shown that improved antioxidative capacity of high-density lipoprotein (HDL) reduces macrophage recruitment, with reduced plaque load within a short time frame by increasing platelet activating factor (PAF) acetyl hydrolase activity or increasing HDL itself (Theilmeier et al., 2000; Shah et al., 2001). We have, in addition, recently demonstrated that 80 mg/kg body weight atorvastatin administered for a very short time can rapidly reduce macrophage content of plaques in apolipoprotein-E-knockout (ApoE-KO) mice (Larmann et al., 2013). In patients at high risk of myocardial infarction without imminent surgery, short-term statin treatment affords protection against myocardial infarction (Patti et al., 2011). In clinical studies testing statin therapy for patients at cardiovascular risk undergoing surgery, the incidence of myocardial infarction and perioperative mortality is reduced (Liakopoulos et al., 2008). Preoperative interleukin-6 (IL-6) plasma levels are associated with myocardial infarction and are reduced in patients receiving statins (van der Meij et al., 2013).

**TRANSLATIONAL IMPACT**

**Clinical issue**

Perioperative myocardial infarction (PMI) is still one of the most dangerous complications for individuals undergoing any type of surgery and has a strong influence on mortality. Not only can individuals who are diagnosed with severe cardiovascular disease prior to surgery suffer from PMI, but also those whose atherosclerotic lesions have remained silent up until the surgical procedure can be at risk. Strategies for prevention and short-term pharmaceutical interventions are rare. This is at least in part due to the lack of animal models for complex atherosclerotic plaques that would allow testing of new interventions.

**Results**

In this study, the authors used apolipoprotein-E-knockout (ApoE-KO) mice (which are prone to develop atherosclerosis) and fed them with a high-cholesterol diet for 7 weeks. Then, the animals were exposed to perioperative stress consisting of general anesthesia, laparotomy and mild blood loss. This procedure led to an increase in atherosclerotic plaque volume and in complexity of the lesions in these mice. A short-term intervention with high-dose atorvastatin (an effective preventive strategy used in humans) led to smaller volume and more stable lesions. Because surgery always induces an inflammatory response, the authors showed that blocking interleukin-6 but not its trans-signaling pathway also leads to stabilization of plaques and reduces plaque volume.

**Implications and future directions**

This study developed a model of perioperative stress in mice prone to atherosclerosis that mimics a standard surgical procedure taking place hundreds of thousands of times every day worldwide. A huge number of individuals who undergo surgery succumb to PMI every year. The current model is not only able to show the actual impact of perioperative stress on atherosclerosis but it can also be used to test perioperative prevention strategies. Future potential interventions that could be tested include beta-blocker treatment, the use of engineered lipoproteins that prevent lipoprotein accumulation and atherosclerotic plaque formation, and pharmacological strategies to stabilize plaques.

Mouse models to study perioperative stress and its effect on plaque growth and stability are currently not available. Reliable models for plaque complexity, growth or rupture have been unavailable in mice (Bennett, 2002; Cullen et al., 2003). Johnson and colleagues, however, reported that, in ApoE-KO mice, rather complex lesions with spontaneous disruption of the luminal plaque surface and overgrowth of plaque material over these areas specifically develop in the innominate artery during the last week of 8 weeks of Western diet (Johnson et al., 2005). The presence of ruptured plaques strictly depends on the duration of the diet, with a sharp increase in the incidence of specific features for plaque rupture at the end of the diet. Other sites of the vascular tree were unaffected.

We reasoned that, if perioperative stress contributes to growth of plaques and affects their stability, this should be detectable in lesions present in the innominate artery. We therefore first developed and characterized a double-hit model of perioperative plaque growth and stability mimicking perioperative stress through the combination of a laparotomy with acute non-replaced blood loss of approximately 20% of total blood volume in general anesthesia. We further tested whether acute high-dose statin therapy or inhibition of IL-6-mediated inflammation would reduce plaque growth and affect their stability in this model.

## RESULTS

### Lipoprotein profiles

**Fig. 1.**
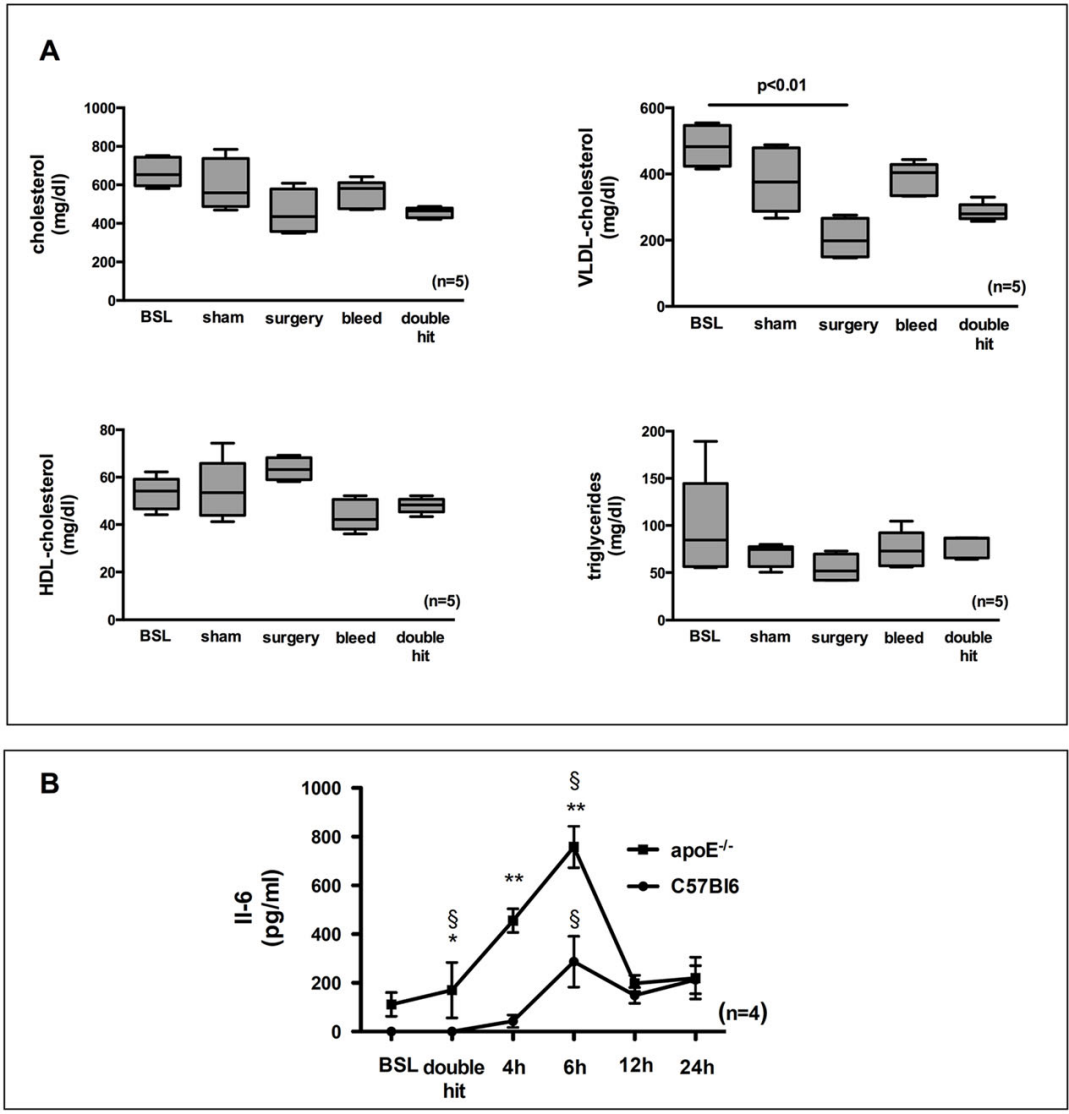
**Lipoprotein profiles and plasma IL-6 levels in C57BL6/J animals and in ApoE-deficient animals on a Western diet subjected to the combination of surgery and blood loss (double hit).** (A) For baseline (BSL) measurements, blood was drawn from the retrobulbar plexus without any intervention and lipoprotein profiles were measured. Measurements for the other groups took place after the procedure. There were no differences in any of the components except for a decrease in VLDL cholesterol levels in the surgery group. Kruskal–Wallis and Dunn's post hoc test, *n*=5 each, *P*<0.01. (B) ELISA for IL-6 detected a significant increase in wild-type mice subjected to the double-hit protocol. Separate animals had to be used to avoid interference with the plaque growth protocol due to additional blood draws. The IL-6 peak occurred at 6 h after procedure. ApoE-deficient mice on a Western diet had slightly higher IL-6 levels compared to the wild types and the IL-6 increase was significantly higher compared to the baseline levels as well as compared to the levels of the wild-type mice. One-way ANOVA and *t*-test, *n*=4, for each time point, *P<0.05; **P<0.01; ^§^*P*<0.05; vs BSL.

We measured plasma lipids in a subgroup of animals to examine the effects of the double hit and its components on lipoprotein profiles. In this context, baseline refers to blood drawn from animals without any procedure on the day of the other groups undergoing the specific interventions. In comparison, sham only underwent general anesthesia, with blood draw taking place 72 h later with the other groups. After 7 weeks on Western diet, baseline animals had similar lipoprotein levels as the sham group. Total cholesterol and HDL remained largely unchanged by any intervention. Surgery alone surprisingly led to a significant decrease of very-low-density lipoprotein (VLDL) cholesterol (*P*<0.05) (Fig. 1A).

### Hemodynamic and inflammatory response to the double hit

Perioperative stress can increase heart rate and blood pressure. Blood pressure remained stable in all groups. No overall differences were detected by the Friedmann test. Within the heart-rate data, significant differences were detected. Although all intervention groups displayed a trend for an increase in heart rate, this difference was significant only after hemorrhage compared to values before intervention (Table 1). Perioperative stress increases plasma levels of IL-6 in patients with atherosclerotic disease (Decker et al., 2005; Winterhalter et al., 2008). ApoE-KO mice had mildly but significantly increased plasma IL-6 compared to C57BL6/J mice before intervention (baseline) (*P*<0.05). When subjected to the double hit, all mice demonstrated a significant elevation of plasma IL-6 levels 6 h thereafter. ApoE-KOs experienced a significantly augmented IL-6 increase compared to their baseline levels and compared to wild-type mice. IL-6 levels returned to baseline within 24 h (Fig. 1B). The acute inflammation translated into an acute phase response as evidenced by an almost 60-fold increase of serum amyloid A (SAA) 24 h after surgery (74.9±76.5 vs 4484±1107 ng/ml, baseline ApoE-KO versus 24 h post double-hit ApoE-KO, *n*=5, *P*<0.01).

**Table 1.**
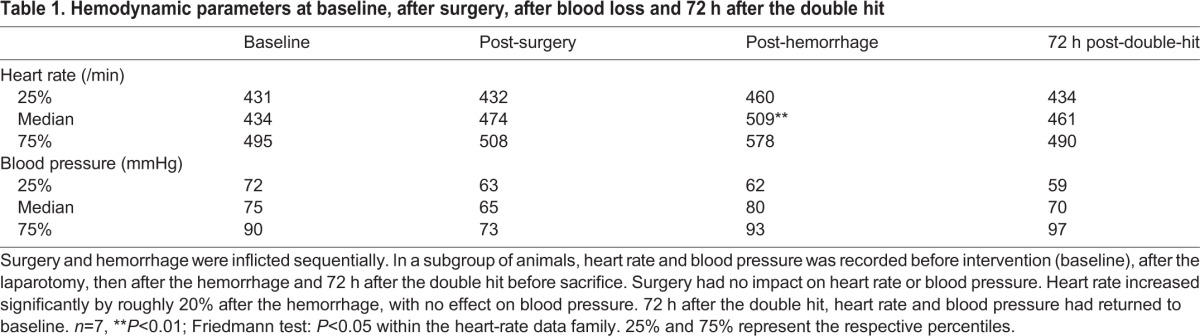
**Hemodynamic parameters at baseline, after surgery, after blood loss and 72 h after the double hit**

### Plaque volume

**Fig. 2.**
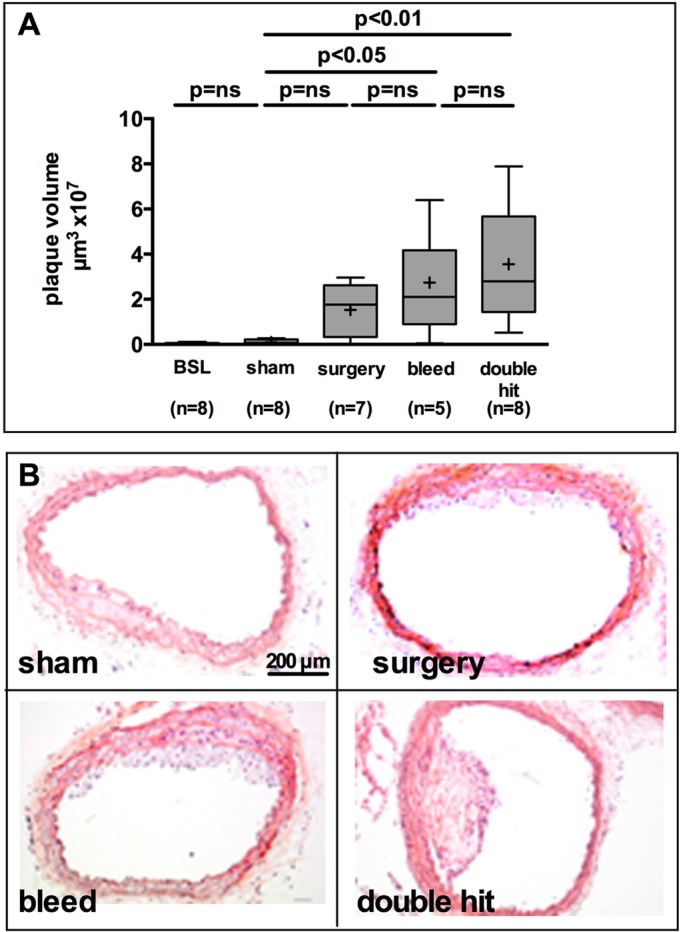
**Plaque volume in atherosclerosis-prone mice increased with perioperative stress.** Matched ApoE-deficient mice of mixed gender, 8 weeks of age, were placed on a Western diet for 7 weeks and subjected to blood loss, laparotomy or the combination thereof to exert perioperative stress (double hit). (A) Plaque area was determined on H&E staining every 42 µm throughout the innominate artery and plaque volume was calculated. Only blood loss or the combination of surgery and blood loss caused a significant increase of plaque volume in the innominate artery. Kruskal–Wallis test. BSL, baseline. (B) Representative micrographs of H&E staining of plaques at the branching of the innominate artery. The overall plaque load was rather small, but eccentric plaques in the double-hit group had complex lesions, whereas animals in the surgery or blood-loss groups had lesions resembling foam cell lesions.

Baseline plaque formation was examined in mice sacrificed after 7 weeks of diet prior to any intervention. Sham animals served as controls for spontaneous plaque growth during the 72 h while interventions took place. Sham mice were sacrificed simultaneously to the single- and double-hit groups. Baseline and sham animals showed no difference in plaque volume. The individual components of the double hit, surgery or blood loss, induced measurable plaque that was nearly absent in sham and baseline animals. Plaque volume in the surgery group was not different from sham animals, but differed significantly between blood-loss and sham mice (*P*<0.05). The double hit induced the most prominent and significant increase in plaque volume compared to the sham group (*P*<0.01) (Fig. 2A,B).

### Plaque composition in mice exposed to perioperative stress

**Fig. 3.**
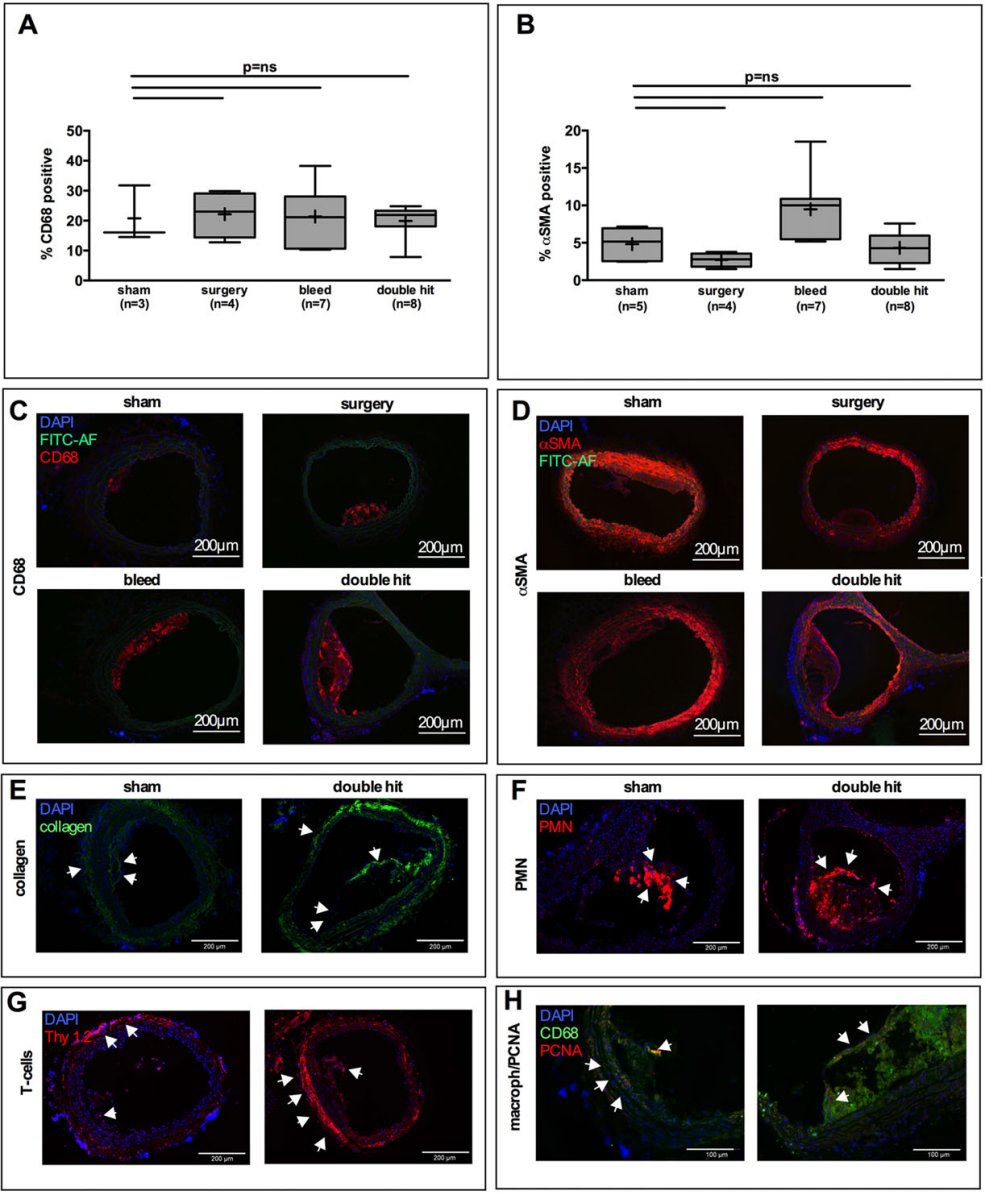
**Composition of plaques.** (A-D) Relative macrophage and smooth muscle cell content did not change in plaques from mice subjected to the combination of surgery and blood loss (double hit). (A,C) Macrophages (CD68) and (B,D) vascular smooth muscle cells (VSMCs; α-smooth muscle actin) were stained by immunofluorescence, and relative areas staining positive for the respective antigens were morphometrically determined. No significant change of the relative contribution of VSMC or macrophages to the larger lesions was detected when double hit was inflicted. Kruskal–Wallis test, sham *n*=3; surgery *n*=4; bleed *n*=7; double hit *n*=8; *n* varies due to differential presence of measureable lesions in groups, *P*=n.s. (E) Collagen content was very low in the plaques and distribution signatures did not overtly differ between groups. (F) Polymorphonuclear (PMN) cell recruitment to the plaques was low. Mainly, PMNs were located in the subendothelial areas likely owing to the acute recruitment of myeloid cells as outlined in the text. (G) T-cell recruitment was mainly observed in the adventitia. Within the plaques, T cells were extremely rare. (H) To assess whether differences in leukocyte proliferation could be responsible for differences in leukocyte content, proliferating cell nuclear antigen (PCNA) was stained. No differences between the groups were detected. Proliferation in the plaques was extremely rare. Arrows indicate typical areas with antigen positive staining.

We then questioned whether plaque composition was altered by perioperative stress and whether smooth muscle cells or macrophages increased preferentially due to the double hit. Macrophage and smooth-muscle-cell markers CD68 and αSMA were therefore assessed. Both CD68 and αSMA content of the plaque, as assessed by the relative area occupied by the respective antigens, remained unchanged by the double-hit intervention (Fig. 3A-D). To further assess differences in plaque composition, collagen was stained. A very inhomogeneous collagen signature and distribution was observed. No qualitative differences were detected (Fig. 3E). To further detail cellular composition, T cells and polymorphonuclear (PMN) cells were stained, showing rather sparse detection of these cell types (Fig. 3F,G). T cells were mainly detectable in the adventitia, whereas PMN-reactive materials were detected in the outer-most layer of the plaques with no differences between sham and double hit. To exclude that local proliferation of macrophages was affected by the interventions, proliferating cell nuclear antigen (PCNA) was stained (Fig. 3H). A very low frequency of proliferating cells was detected in the macrophage-laden plaque areas with likewise no difference between sham and double hit. We concluded that plaque composition was not majorly affected by the double hit and questioned whether plaque stability could still be altered.

### Alterations of plaque stability in mice exposed to perioperative stress

**Fig. 4.**
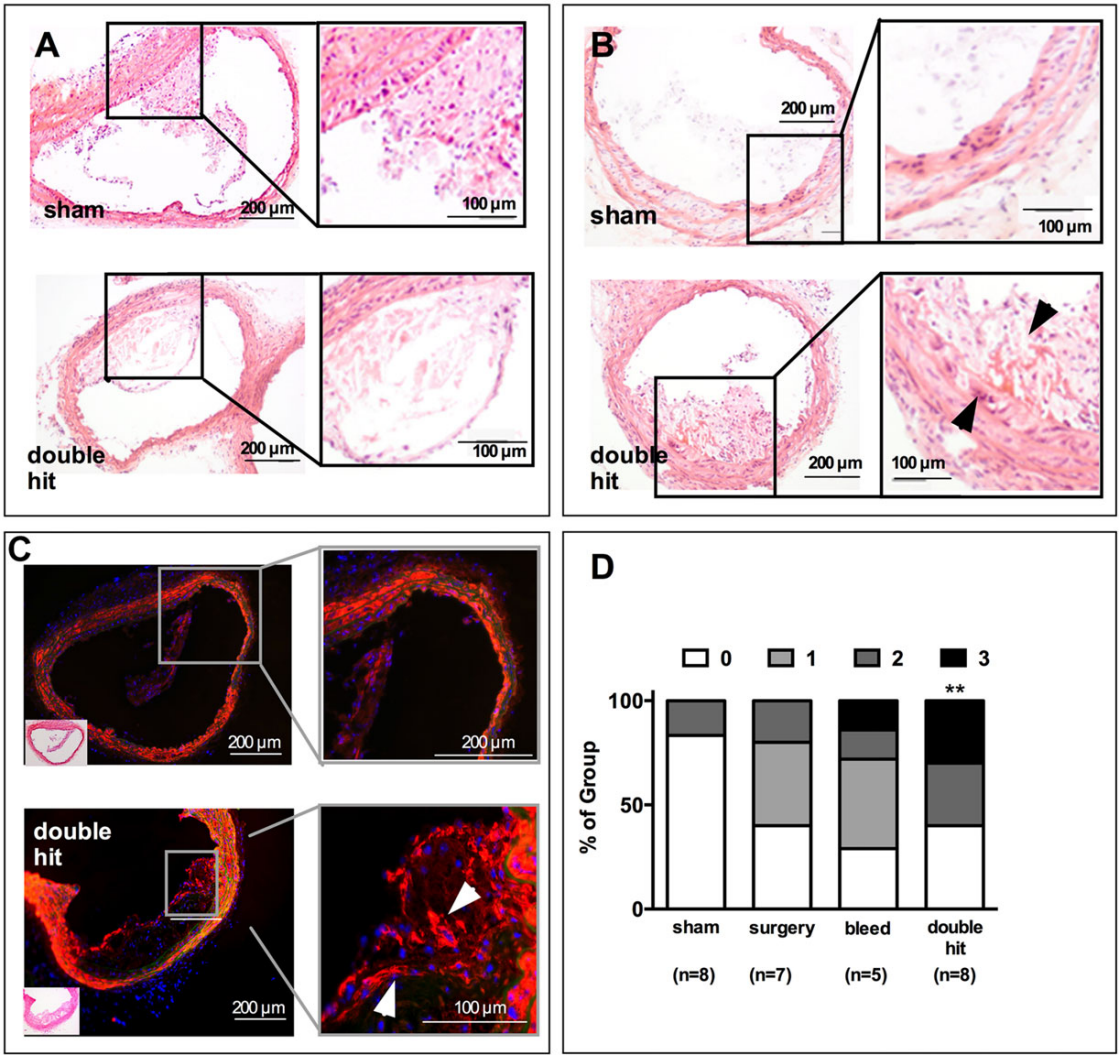
**Signs preceding** **rupture of plaques were detected in animals exposed to the combination of surgery and blood loss (double hit).** (A) A small proportion of lesions in control, but a large proportion of mice exposed to the double hit, demonstrated necrotic areas in the core of the plaque. Such necrosis had to be detectable on at least 50% of the sections to score 1 point. (B) Hemorrhage in the plaque was detected on H&E staining by the presence of red blood cells in the center of the lesion (arrowheads). Signs for intraplaque bleeding had to be present on at least 10% of the analyzed sections that span the whole innominate artery. (C) Buried fibrous caps were defined as αSMA-positive streaks (white arrowheads) that had been overgrown by new plaque material and were interpreted as buried fibrous caps secondary to plaque rupture, reorganization and overgrowth by plaque material. (D) Scoring of the features depicted in A-C with one point each revealed the presence of complex lesions in 60% of the double-hit mice, whereas control mice only had one feature in 32% of the cases, which mostly represented necrotic areas as shown in A. Fisher's exact test, sham, double hit *n*=8; surgery *n*=7; bleed *n*=5; **P<0.01 vs SHAM.

If the double hit led to unstable plaques, more plaques would exhibit necrotic and hemorrhagic areas. The Stary score is commonly used for classification of plaque stability in human atherosclerosis. Stary divides lesions into six different groups by qualitative characteristics such as lipid content, hematoma and layers of fibromuscular tissue (see Table 2 for definitions) (Stary, 2000). We found plaques of group IV to VI mainly in animals exposed to the double hit, whereas mice in the sham, hemorrhage and surgery groups presented less complex plaques, in groups I through III. The Stary score does not, however, provide a sufficiently high resolution of differences in vulnerability, because it collects all complex plaques in class VI, where surface defects, thrombosis and hemorrhage are grouped together. Therefore, we additionally scored the individual components of the Stary score by separately rating the presence or absence of plaque necrosis, intraplaque hemorrhage and buried fibrous caps to generate a gradual score that is accessible to non-parametric statistical analysis. The most stable plaque would have neither necrosis, nor hemorrhage, nor buried fibrous caps (0 points), whereas the most complex lesion would contain all three features (3 points). Mice subjected to the double hit had significantly higher scores, reflecting significantly more complex plaques than sham animals (*P*<0.01) (Fig. 4A-D).

**Table 2.**
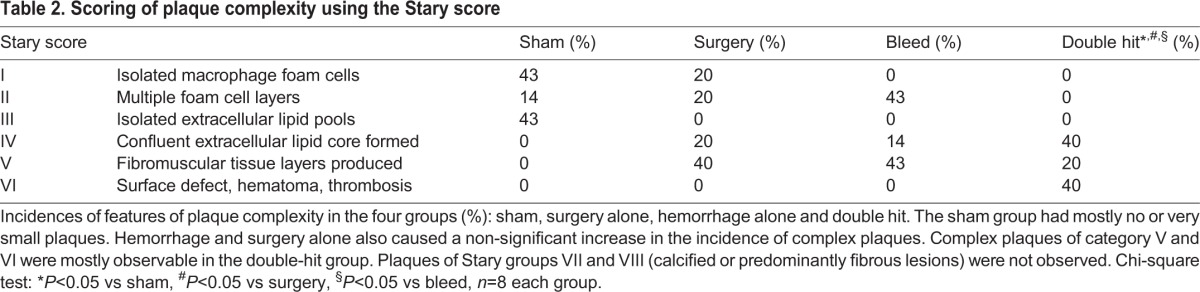
**Scoring of plaque complexity using the Stary score**

### Short-term statin therapy reduces plaque burden and complexity as well as total cholesterol and IL-6

**Fig. 5.**
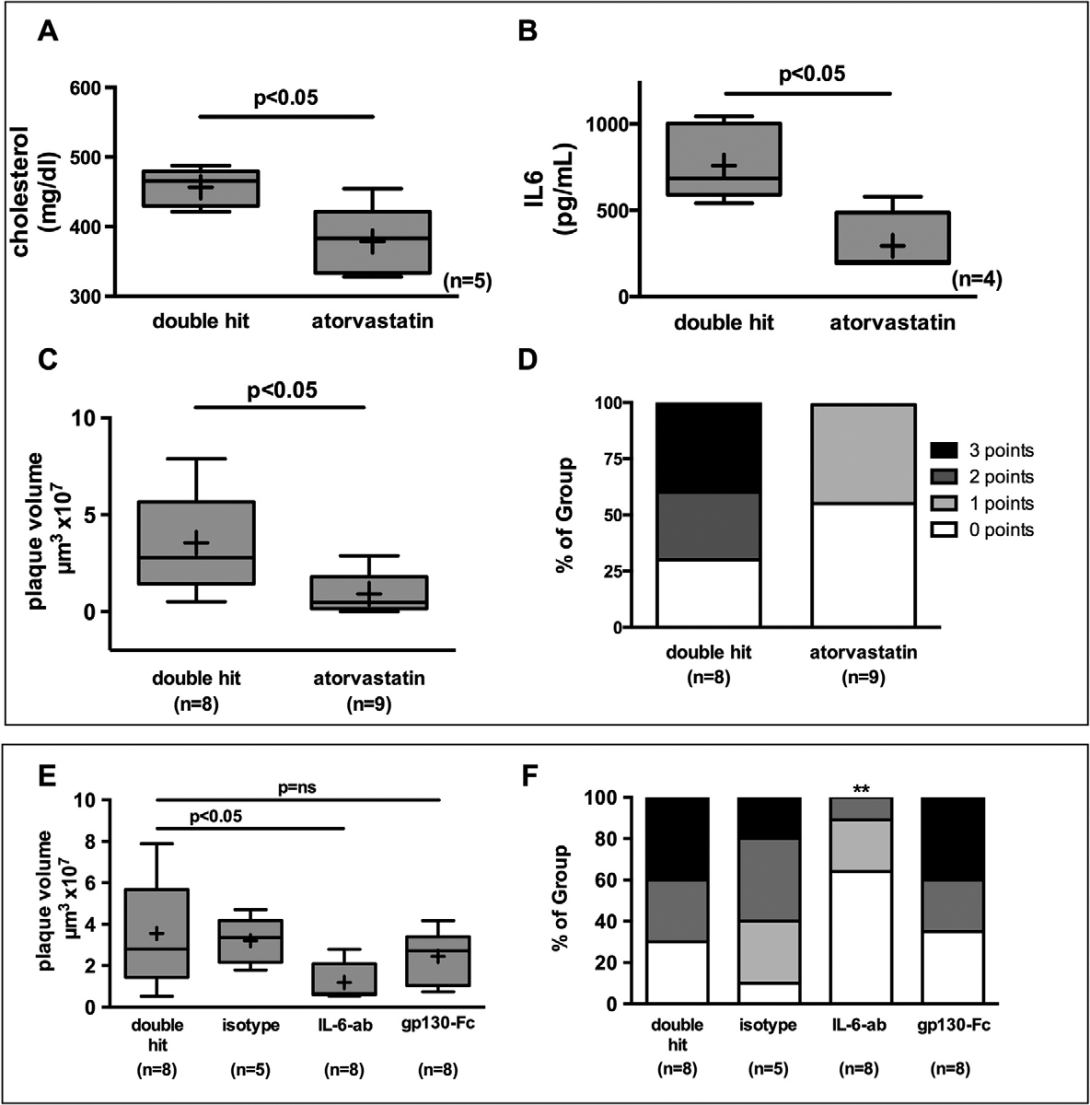
**High-dose statin therapy 3 days prior to and 3 days after surgery in mice exposed to the double hit, and IL-6 signaling pathway interception.** Mice were treated with 80 mg/kg body weight/day atorvastatin, blocking antibodies against IL-6 or the selective inhibition of IL-6 trans-signaling by gp130-Fc by a single subcutaneous injection. (A) Total cholesterol (*n*=5; *P*<0.05), (B) IL-6 plasma levels (*n*=4; *P*<0.05) and (C) plaque volume (double hit *n*=8; atorvastatin *n*=9; *P*<0.05) decreased significantly under atorvastatin. Mann–Whitney *U*-test. (D) Plaque complexity as assessed by the score was likewise significantly reduced (Fisher's exact test, double hit *n*=8; atorvastatin *n*=9; *P*<0.05). (E) The blocking antibody against IL-6 reduced plaque volume, whereas the selective inhibition of IL-6 trans-signaling by gp130-Fc did not yield any reduction in plaque volume. Mann–Whitney *U*-test; double hit, gp130-Fc *n*=8; isotype *n*=5; IL-6 ab *n*=8; *P*<0.05. (F) Plaque complexity was reduced by blocking antibody (Fisher's exact test; double hit, gp130-Fc *n*=8; isotype *n*=5; IL-6 ab *n*=8; **P<0.05 vs double hit).

Statin treatment has been demonstrated to reduce perioperative cardiovascular events in patients at risk (Liakopoulos et al., 2008). To assess whether atorvastatin would exert similar effects in our model, we treated animals with high-dose statin or vehicle 3 days pre- and 3 days postoperatively as previously described (Larmann et al., 2013). Total cholesterol at the time of the double hit (*P*<0.05) and serum IL-6 level at the 6-h time point were decreased (*P*<0.05; Fig. 5A,B). Atorvastatin significantly reduced plaque volume compared to untreated animals (*P*<0.05; Fig. 5C). Lesions from statin-treated mice also reached significantly fewer points in the plaque score, indicating successful prevention of double-hit-induced plaque instability (*P*<0.05) (Fig. 5D).

### Blocking IL-6 signaling reduces plaque burden and complexity

IL-6 signaling can foster local inflammation by direct activation of the classical IL-6 receptor as well as systemic inflammation by trans-signaling through IL-6 complexed to its soluble receptor, which then engages gp130 (Schuett et al., 2012). Treatment with an antibody blocking IL-6 effectively reduced the increase in plaque volume and plaque complexity (*P*<0.05). Blocking of the trans-signaling pathway by the fusion protein gp130-FC had no effect on the double-hit-induced increase in plaque volume or the morphology score. These findings indicate that systemic, likely hepatically induced, inflammation is signaled through IL-6. IL-6 then engaged its classical receptor and contributed to plaque growth in response to hemorrhage and surgery (Fig. 5E,F).

## DISCUSSION

Coronary artery disease is the underlying disease of perioperative myocardial infarctions (Landesberg et al., 2009; Priebe, 2011). The effect of perioperative stress on plaque growth and stability remains incompletely understood. Only very few strategies are currently available to reduce the perioperative cardiovascular risk (Fleisher and Eagle, 2001).

We report here the development of a mouse model to assess perioperative growth and stability of atherosclerotic lesions. A combination of surgery and blood loss drives plaque growth with unaltered cellular plaque composition in ApoE-KO mice. The animals mounted a mild hemodynamic response to the double hit. The double hit had no meaningful effects on atherogenic lipoprotein levels. Surgery and hemorrhage prompted an increase in circulating pro-inflammatory IL-6. Vulnerable and ruptured plaques were more prevalent in mice exposed to the double hit compared to sham or single-hit groups. High-dose, short-term atorvastatin treatment lowered total cholesterol, reduced the release of pro-inflammatory IL-6, prevented plaque growth and reduced plaque complexity. Inhibition of classical but not trans-signaling of IL-6 reduced plaque growth and signs of instability.

Surgical procedures exert postoperative stress that can precipitate myocardial infarction (Mangano, 2004; Decker et al., 2005; Winterhalter et al., 2008). About half of perioperative myocardial infarctions occur on the basis of supply-demand mismatches due to increases in heart rate and myocardial wall stress, whereas the other half is accounted for by plaque rupture (Dawood et al., 1996; Cohen and Aretz, 1999; Kertai et al., 2003; Priebe, 2011; Hanson et al., 2013). Plaque rupture and growth have been closely linked to inflammation. The inflammatory response to surgery has been intensely investigated (Anand et al., 1990; Anand and Hickey, 1992; Chew et al., 2001; Larmann and Theilmeier, 2004; Kohl and Deutschman, 2006). There is a large number of surgery-, trauma- and hemorrhage-induced stress models in small rodents that have been used to elucidate the inflammatory response; however, the effects of the surgical, traumatic or hemorrhage-induced stress response on volume and stability of atherosclerotic lesions have, to date, not been examined (Frink et al., 2011). On the other hand, the effect of chronic inflammatory stressors on the precipitation of cardiovascular events has been well characterized (Nawrot et al., 2011). Whether surgical procedures have an impact on plaque has not been thoroughly investigated, mainly because of the lack of relevant animal models.

Therefore, we devised a double-hit model exposing mice prone to develop atherosclerosis (Plump et al., 1992) to a surgical procedure of medium severity combined with a substantial, but not severe, blood loss of 400 µl, which we estimated to be 20% of total blood volume (Wu et al., 1981). We chose to employ a surgical procedure that was organ-independent to render the results generalizable to other surgical procedures. We cannot exclude that particular procedures combined with certain concomitant diseases will have more or less effect on atherosclerotic lesions (Pearse et al., 2012). The combination with hemorrhage could raise the concern that the inflammatory response might be mainly driven by this component. The interventions induced an increase in heart rate that indicated hemodynamic strain that was not in the range of a hemorrhagic shock (Lonati et al., 2012).

Inflammation and stress have been reported to cause alterations in lipoprotein levels (Carpentier and Scruel, 2002). In our model, alterations of atherogenic lipoproteins were mild and non-significant except for a decrease of VLDL cholesterol in the surgery-only group, which we cannot explain. HDL or triglyceride levels were unaffected. Therefore, the increase in plaque volume was not due to the altered lipoprotein profiles.

The assumption that atherosclerosis has only slow dynamics in lesional morphology (Ross, 1999; Plump et al., 1992) has been challenged (Theilmeier et al., 2000; Shah et al., 2001; Larmann et al., 2013). Fisher, Randolph and colleagues, using a transplant model (Llodra et al., 2004), suggested active recirculation of macrophages and dynamic changes of plaque composition (Feig et al., 2009). In the double-hit group, lesion size increased. The relative content of macrophages and VSMCs in the plaques was not, however, changed by the double hit, suggesting that a net increase in the recruitment of both cell types occurred during the 72 h following the insult.

In our animals, the double hit moved plaques to higher complexity groups in the Stary score. This difference was more apparent in our newly developed score that more closely depicts individual features of plaque complexity, suggesting that the rapid growth of the plaques is associated with an increased risk for plaque complexity (Johnson and Jackson, 2001; Bea et al., 2002; Rattazzi et al., 2005; Sasaki et al., 2006). Meanwhile, the presence of complex plaques in the innominate artery of ApoE-deficient mice, on an atherogenic diet, is well accepted and widely used as a model for human plaque vulnerability (Matoba et al., 2013). At the same time caution is obligatory in extrapolating to human plaque. We do however conclude from our data that mild surgical stress combined with hemorrhage can inflict growth of plaques with signs of instability in previously hardly diseased vessels.

IL-6 plays a pivotal role in growth of atherosclerotic plaques. IL-6 levels are increased in patients with increased atherosclerotic plaque load (Willerson and Ridker, 2004). When patients with atherosclerotic burden underwent short-term statin treatment, IL-6 levels were decreased, and myocardial infarction and death were reduced (Liakopoulos et al., 2008; van der Meij et al., 2013). The humoral inflammatory response to the double hit was significantly amplified in Western-diet-fed ApoE-deficient animals. The trans-signaling pathway of IL-6 importantly contributes to the promotion of plaque growth (Huber et al., 1999; Schieffer et al., 2004; Luchtefeld et al., 2007; Schuett et al., 2012). An IL-6-neutralizing antibody reduced plaque volume and complexity, whereas selective inhibition of trans-signaling with gp130-Fc did not. We hypothesize that IL-6 neutralization suppressed the acute phase reaction (Menger and Vollmar, 2004).

Short-term, high-dose atorvastatin treatment reduces macrophage content of established lesions (Larmann et al., 2010). Atorvastatin at 80 mg/kg body weight significantly reduced total cholesterol, plasma IL-6, plaque growth and signs of instability in this novel model. Total cholesterol was decreased in statin-treated animals, which could have contributed to plaque stabilization. The prominent decrease in IL-6 in statin-treated mice suggests additional pleiotropic effects (Werner et al., 2002). The statin effect might thus also be fostered by a reduction of macrophage recruitment to the plaque, because plaque composition did not change, whereas plaque volume was significantly reduced (Potteaux et al., 2011).

Our study has several limitations. Our surgical procedure consisted of opening and closing the abdominal cavity. This renders the model unspecific but very well generalizable to any surgical trauma. Also, the bloodshed was not replaced as it would be in clinical situations. However, our double hit induced no shock but rather mild hemodynamic strain, which is highly compatible with the clinical scenario of major surgery. At the time when the double hit was inflicted, the plaque volume in baseline and sham animals was small. We can, therefore, not extrapolate to the effects of surgery and hemorrhage or the effect of statins on pre-existing advanced lesions.

Nevertheless, we do provide evidence that a short period of perioperative stress can induce significant plaque growth and that these plaques exhibit signs of plaque rupture. A brief treatment with high-dose statin can reduce plaque growth as well as complexity. Inhibition of IL-6, but not its trans-signaling, prevented plaque growth and decreased complexity. In conclusion, we have developed a useful model to study the pathophysiology of perioperative plaque dynamics, which was subsequently used to prove perioperative statin therapy and IL-6 inhibition to be useful, and which could serve to test the efficacy of plaque stabilizing strategies in the future.

## MATERIALS AND METHODS

### Animals

Animal studies were approved by the Institutional Review Board and the regional authorities [Niedersächsisches Landesamt für Verbrauchershcutz und Lebensmittelsicherheit (LAVES)]. Animals were handled according to the Guide for the Care and Use of Laboratory Animals published by the National Academy of Science. In total, 7 male C57BL6/J and 66 congenic ApoE-deficient mice (Plump et al., 1992), 8 weeks of age (*ApoE*^−/−^; 30 females, 36 males; 23±1 g body weight; Jackson Laboratories, Bar Harbor, Maine, USA), were fed a high-cholesterol diet containing 1.25% cholesterol for 7 weeks (Altromin, Lage, Germany) and assigned to groups to achieve the best age- and gender-match possible in a non-randomized, non-blinded fashion. All analyses were carried out in a blinded fashion and by three examiners as indicated where judgment and scoring was performed.

### Double-hit model

After 7 weeks of Western diet, mice were subjected to a double-hit perioperative stress model. Surgical procedures exert stress due to, among other factors, a combination of surgical injury and major fluid shifts. The surgical procedure was therefore mimicked by a longitudinal median laparotomy in general anesthesia with 1.5 vol% Isoflurane in 100% oxygen lasting 30 min. The abdomen was closed with single-knot sutures. Blood loss was inflicted to reduce blood volume by roughly 20%. 400 µl blood was drawn from the retrobulbar venous plexus (*n*=8). Blood pressure and heart rate were assessed in selected animals (*n*=7) before and after the double-hit model was inflicted as well as at the time of sacrifice using a tail cuff device (NIBP System, Powerlab, AD Instruments, Marburg, Germany). The effects of laparotomy and blood loss were independently examined in two subgroups (surgery *n*=7; bleed *n*=5) to establish their individual contribution to the double-hit model. Baseline animals (*n*=8) were sacrificed after 7 weeks of diet without further intervention to assess spontaneous plaque rupture during that time. Sham animals (*n*=8) were subjected to 30 min of general anesthesia with 1.5 vol% Isoflurane in 100% oxygen at the same time of surgery as the double-hit group and sacrificed simultaneously with the intervention groups 3 days later.

In a subgroup of animals (ApoE-KO *n*=4; C57BL6/J, wild type *n*=5), blood (100 µl) was collected 1 week before perioperative stress was induced by the double hit. For determination of plasma IL-6, 4 and 12 or 6 and 24 h thereafter (at maximum two extra blood draws) blood was collected from both genotypes, gently mixed with citrate, and plasma was centrifuged and stored frozen at −80°C. These animals were excluded from the analysis of plaque volume to avoid bias by the additional impact on plaque stability due to the extra blood loss and procedures.

A separate group of mice (*n*=9) was treated with atorvastatin 80 mg/kg body weight per day, administered orally through a gastric tube starting 72 h before surgery until sacrifice 72 h post-surgery. Another group of animals was treated with IL-6-blocking antibody (*n*=8; 200 µg subcutaneously, clone MP5-20F3 BD Pharmingen, Heidelberg, Germany), the respective isotype control (*n*=5; 200 µg subcutaneously, rat IgG1, BD Pharmingen, Heidelberg, Germany) or the fusion protein gp130-Fc (*n*=8; 200 µg subcutaneously, R&D systems, Art-no 468-MG-100, Minneapolis, USA) by a single subcutaneous injection after the double hit had been inflicted. Animals were re-anesthetized 72 h after surgery, euthanized by exsanguination from the caval vein and perfused from a left ventricular puncture using 0.9% saline at physiological pressure. The innominate arteries were dissected and cryoembedded in OCT for histology and immunohistochemistry.

### Lipoprotein profiles

In plasma samples, total cholesterol, triglycerides (Roche Molecular Biochemicals, Mannheim, Germany) and phospholipids (Wako, Neuss, Germany) were determined enzymatically using commercially available kits (*n*=5 per group). Not all animals yielded sufficient amounts of blood for parallel assessment of IL-6 and lipoprotein profiles. Animals with sufficient plasma sample volume were selected. The cholesterol content within different lipoprotein subclasses from individual plasma samples was determined following sequential tabletop ultracentrifugation as published (Tietge et al., 2000). Cholesterol concentrations within each fraction were measured as detailed above.

### ELISA

Concentrations of IL-6 and SAA were assayed in plasma of wild-type and ApoE-KO mice at the time points indicated above and were determined using ELISA kits for IL-6 (BE45061, IBL International, Hamburg, Germany) and SAA (Tridelta Development Ltd, Maynooth, Ireland) according to the protocol of the manufacturers.

### Histology and immunohistochemistry

The innominate artery was completely cryosectioned at a thickness of 7 µm. Lesion size, quantified on hematoxylin and eosin (H&E) stainings, was assessed on every sixth section throughout the innominate artery (immediately distal of aortic origin to immediately proximal of the bifurcation of the innominate artery), rendering the distance between two analyzed sections to be 42 µm. Plaque volume (µm^3^) was calculated as Σ_total plaque volume_=(plaque area*_n_*×42 µm)+(plaque area*_n_*_-1_×42 µm)+⋯+(plaque area*_n_*_-*n*_×42 µm). *n* was determined by the length of the innominate artery, with up to 25 sections carrying plaque. Animals with sections showing cutting artifacts in two consecutive sections were completely excluded from the analysis (<10%); distribution across groups was even.

Adjacent sections were stained for macrophages (rat anti-mouse CD68, clone FA-11, Serotec, Düsseldorf, Germany) or VSMCs (α-smooth muscle actin; αSMA, clone 1A4, Sigma, St Louis, USA). For detection, a Cy3-coupled secondary antibody (goat anti-rat and goat anti-mouse, Jackson ImmunoResearch, West Grove, USA) was used. The primary mouse antibody (αSMA) was developed using a mouse-on-mouse detection kit. Micrographs to assess macrophage and VSMC content were taken using an Olympus IX81 microscope (Olympus Europa Holding GmbH, Hamburg, Germany). Morphometry was performed using CellF life science fluorescence imaging software (version 3.1, Olympus, Hamburg, Germany). Macrophage and VSMC content are expressed as the percentage of the lesion staining positive for macrophages or VSMCs.

### Morphology score

To assess plaque complexity and/or vulnerability, plaques were scored following the Stary criteria (Table 2) (Stary, 2000). Because the Stary score collects complex plaques in one single category and because the categorization is mathematically not accessible for comparisons, we decided to additionally and individually score the features of the Stary score that indicate plaque complexity, such as necrosis, hemorrhage and buried fibrous caps. Similar scoring strategies were used in the literature (Johnson and Jackson, 2001). These data were consolidated in a novel score by assigning scoring points for the presence of each individual feature as assessed on H&E and αSMA stainings. Necrotic core was detected when central or basal and hematoxylin-free areas of the plaque contained debris or lipids. A point for necrosis was only assigned when a necrotic core was visible on at least 50% of the examined sections to avoid misinterpretation of cutting artifacts and to assure a minimum size of necrosis leading to a scoring point. Intraplaque hemorrhage was defined as clusters of erythrocytes present in the plaque interior on H&E-stained sections and red blood cell autofluorescence was visible in the FITC channel. Buried fibrous caps were detected as clusters of cap-like organized VSMCs within the plaque that were covered by newly formed plaque on any of the analyzed sections stained for αSMA. Each section was evaluated by three different blinded assessors (H.J., S.C., G.S.) and discrepancies in the score assignment were solved by consensus in each case.

For the presence of each feature, one scoring point was assigned and added to calculate the score. At least seven sections per animal were used for scoring plaque complexity. Data are presented as the percentage of animals in every group reaching the respective value. Mice without plaque were assigned a score of 0.

### Statistical analysis

Data were not normally distributed. Therefore, data are presented as median (horizontal line), mean (+), 25/75% boxes and 5%/95% whiskers. Mann–Whitney *U*-test was carried out to identify differences between groups. Whenever more than two groups were compared, Kruskal–Wallis test was employed for global assessment of differences in the data family before limited groupwise comparisons were done using Dunn's test to correct for multiple testing. Incidences of plaque complexity features were compared using chi-square tests. When more than two groups were compared, Holm's correction was carried out. Changes of hemodynamics over time were compared using Friedmann's test followed by a groupwise post test. The data pertaining to IL-6 levels were normally distributed (Kolmogorov–Smirnoff) and subjected to ANOVA, and groupwise comparisons were carried out by Bonferroni-corrected *t*-test (Prism, GraphPad Prism).

## References

[DMM018713C1] Anand K. J. S. and Hickey P. R. (1992). Halothane-morphine compared with high-dose sufentanil for anesthesia and postoperative analgesia in neonatal cardiac surgery. *N. Engl. J. Med.* 326, 1-9. 10.1056/NEJM199201023260101 1530752

[DMM018713C2] Anand K. J. S., Hansen D. D. and Hickey P. R. (1990). Hormonal-metabolic stress responses in neonates undergoing cardiac surgery. *Anesthesiology* 73, 661-670. 10.1097/00000542-199010000-00012 2221435

[DMM018713C3] Bea F., Blessing E., Bennett B., Levitz M., Wallace E. P. and Rosenfeld M. E. (2002). Simvastatin promotes atherosclerotic plaque stability in apoE-deficient mice independently of lipid lowering. *Arterioscler. Thromb. Vasc. Biol.* 22, 1832-1837. 10.1161/01.ATV.0000036081.01231.16 12426212

[DMM018713C4] Bennett M. R. (2002). Breaking the plaque: evidence for plaque rupture in animal models of atherosclerosis. *Arterioscler. Thromb. Vasc. Biol.* 22, 713-714. 10.1161/01.ATV.0000019008.18226.C3 12006380

[DMM018713C5] Carpentier Y. A. and Scruel O. (2002). Changes in the concentration and composition of plasma lipoproteins during the acute phase response. *Curr. Opin. Clin. Nutr. Metab. Care* 5, 153-158. 10.1097/00075197-200203000-00006 11844981

[DMM018713C6] Chew M. S., Brandslund I., Brix-Christensen V., Ravn H. B., Hjortdal V. E., Pedersen J., Hjortdal K., Hansen O. K. and Tønnesen E. (2001). Tissue injury and the inflammatory response to pediatric cardiac surgery with cardiopulmonary bypass: a descriptive study. *Anesthesiology* 94, 745-753; discussion 745A 10.1097/00000542-200105000-00010 11388523

[DMM018713C7] Cohen M. C. and Aretz T. H. (1999). Histological analysis of coronary artery lesions in fatal postoperative myocardial infarction. *Cardiovasc. Pathol.* 8, 133-139. 10.1016/S1054-8807(98)00032-5 10722235

[DMM018713C8] Cullen P., Baetta R., Bellosta S., Bernini F., Chinetti G., Cignarella A., von Eckardstein A., Exley A., Goddard M., Hofker M. et al. (2003). Rupture of the atherosclerotic plaque: does a good animal model exist? *Arterioscler. Thromb. Vasc. Biol.* 23, 535-542. 10.1161/01.ATV.0000060200.73623.F8 12615660

[DMM018713C9] Dawood M. M., Gutpa D. K., Southern J., Walia A., Atkinson J. B. and Eagle K. A. (1996). Pathology of fatal perioperative myocardial infarction: implications regarding pathophysiology and prevention. *Int. J. Cardiol.* 57, 37-44. 10.1016/S0167-5273(96)02769-6 8960941

[DMM018713C10] Decker D., Tolba R., Springer W., Lauschke H., Hirner A. and von Ruecker A. (2005). Abdominal surgical interventions: local and systemic consequences for the immune system--a prospective study on elective gastrointestinal surgery. *J. Surg. Res.* 126, 12-18. 10.1016/j.jss.2005.01.006 15916969

[DMM018713C11] Feig J. E., Quick J. S. and Fisher E. A. (2009). The role of a murine transplantation model of atherosclerosis regression in drug discovery. *Curr. Opin. Investig. Drugs* 10, 232-238. PMC4662935 19333880

[DMM018713C12] Fleisher L. A. and Eagle K. A. (2001). Lowering cardiac risk in noncardiac surgery. *N. Engl. J. Med.* 345, 1677-1682. 10.1056/NEJMcp002842 11759647

[DMM018713C13] Frink M., Andruszkow H., Zeckey C., Krettek C. and Hildebrand F. (2011). Experimental trauma models: an update. *J. Biomed. Biotechnol.* 2011, 797383 10.1155/2011/797383 21331361 PMC3035380

[DMM018713C14] Gualandro D. M., Campos C. A., Calderaro D., Yu P. C., Marques A. C., Pastana A. F., Lemos P. A. and Caramelli B. (2012). Coronary plaque rupture in patients with myocardial infarction after noncardiac surgery: frequent and dangerous. *Atherosclerosis* 222, 191-195. 10.1016/j.atherosclerosis.2012.02.021 22410124

[DMM018713C15] Hanson I., Kahn J., Dixon S. and Goldstein J. (2013). Angiographic and clinical characteristics of type 1 versus type 2 perioperative myocardial infarction. *Catheter Cardiovasc. Interv.* 82, 622-628. 10.1002/ccd.24626 22926992

[DMM018713C16] Huber S. A., Sakkinen P., Conze D., Hardin N. and Tracy R. (1999). Interleukin-6 exacerbates early atherosclerosis in mice. *Arterioscler. Thromb. Vasc. Biol.* 19, 2364-2367. 10.1161/01.ATV.19.10.2364 10521365

[DMM018713C17] Johnson J. L. and Jackson C. L. (2001). Atherosclerotic plaque rupture in the apolipoprotein E knockout mouse. *Atherosclerosis* 154, 399-406. 10.1016/S0021-9150(00)00515-3 11166772

[DMM018713C18] Johnson J., Carson K., Williams H., Karanam S., Newby A., Angelini G., George S. and Jackson C. (2005). Plaque rupture after short periods of fat feeding in the apolipoprotein E-knockout mouse: model characterization and effects of pravastatin treatment. *Circulation* 111, 1422-1430. 10.1161/01.CIR.0000158435.98035.8D 15781753

[DMM018713C19] Kamel H., Johnston S. C., Kirkham J. C., Turner C. G., Kizer J. R., Devereux R. B. and Iadecola C. (2012). Association between major perioperative hemorrhage and stroke or Q-wave myocardial infarction. *Circulation* 126, 207-212. 10.1161/CIRCULATIONAHA.112.094326 22679143 PMC3986632

[DMM018713C20] Kertai M. D., Klein J., van Urk H., Bax J. J. and Poldermans D. (2003). Cardiac complications after elective major vascular surgery. *Acta Anaesthesiol. Scand.* 47, 643-654. 10.1034/j.1399-6576.2003.00149.x 12803580

[DMM018713C21] Kohl B. A. and Deutschman C. S. (2006). The inflammatory response to surgery and trauma. *Curr. Opin. Crit. Care* 12, 325-332. 10.1097/01.ccx.0000235210.85073.fc 16810043

[DMM018713C22] Landesberg G., Beattie W. S., Mosseri M., Jaffe A. S. and Alpert J. S. (2009). Perioperative myocardial infarction. *Circulation* 119, 2936-2944. 10.1161/CIRCULATIONAHA.108.828228 19506125

[DMM018713C23] Larmann J. and Theilmeier G. (2004). Inflammatory response to cardiac surgery: cardiopulmonary bypass versus non-cardiopulmonary bypass surgery. *Best Practice Res.* 18, 425-438. 10.1016/j.bpa.2003.12.004 15212337

[DMM018713C24] Larmann J., Frenzel T., Hahnenkamp A., Herzog C., Lorenz A., Steinbicker A. U., Calmer S., Harendza T., Schmitz M., Echtermeyer F. et al. (2010). In vivo fluorescence-mediated tomography for quantification of urokinase receptor-dependent leukocyte trafficking in inflammation. *Anesthesiology* 113, 610-618. 10.1097/aln.0b013e3181e99bfc 20693875

[DMM018713C25] Larmann J., Frenzel T., Schmitz M., Hahnenkamp A., Demmer P., Immenschuh S., Tietge U. J. F., Bremer C. and Theilmeier G. (2013). In vivo fluorescence-mediated tomography imaging demonstrates atorvastatin-mediated reduction of lesion macrophages in ApoE−/− mice. *Anesthesiology* 119, 129-141. 10.1097/ALN.0b013e318291c18b 23559030

[DMM018713C26] Liakopoulos O. J., Choi Y.-H., Haldenwang P. L., Strauch J., Wittwer T., Dorge H., Stamm C., Wassmer G. and Wahlers T. (2008). Impact of preoperative statin therapy on adverse postoperative outcomes in patients undergoing cardiac surgery: a meta-analysis of over 30,000 patients. *Eur. Heart J.* 29, 1548-1559. 10.1093/eurheartj/ehn198 18506053

[DMM018713C27] Llodra J., Angeli V., Liu J., Trogan E., Fisher E. A. and Randolph G. J. (2004). Emigration of monocyte-derived cells from atherosclerotic lesions characterizes regressive, but not progressive, plaques. *Proc. Natl. Acad. Sci. USA* 101, 11779-11784. 10.1073/pnas.0403259101 15280540 PMC511052

[DMM018713C28] Lonati C., Sordi A., Giuliani D., Spaccapelo L., Leonardi P., Carlin A., Ottani A., Galantucci M., Grieco P., Catania A. et al. (2012). Molecular changes induced in rat liver by hemorrhage and effects of melanocortin treatment. *Anesthesiology* 116, 692-700. 10.1097/ALN.0b013e318246ea68 22266570

[DMM018713C29] Luchtefeld M., Schunkert H., Stoll M., Selle T., Lorier R., Grote K., Sagebiel C., Jagavelu K., Tietge U. J. F., Assmus U. et al. (2007). Signal transducer of inflammation gp130 modulates atherosclerosis in mice and man. *J. Exp. Med.* 204, 1935-1944. 10.1084/jem.20070120 17664290 PMC2118681

[DMM018713C30] Mangano D. T. (2004). Perioperative medicine: NHLBI working group deliberations and recommendations. *J. Cardiothorac. Vasc. Anesthesia* 18, 1-6. 10.1053/j.jvca.2003.10.002 14973791

[DMM018713C31] Matoba T., Sato K. and Egashira K. (2013). Mouse models of plaque rupture. *Curr. Opin. Lipidol.* 24, 419-425. 10.1097/mol.0b013e3283646e4d 23942269

[DMM018713C32] Menger M. D. and Vollmar B. (2004). Surgical trauma: hyperinflammation versus immunosuppression? *Langenbecks Arch. Surg.* 389, 475-484. 10.1007/s00423-004-0472-0 15173946

[DMM018713C33] Nawrot T. S., Perez L., Künzli N., Munters E. and Nemery B. (2011). Public health importance of triggers of myocardial infarction: a comparative risk assessment. *Lancet* 377, 732-740. 10.1016/S0140-6736(10)62296-9 21353301

[DMM018713C34] Patti G., Cannon C. P., Murphy S. A., Mega S., Pasceri V., Briguori C., Colombo A., Yun K. H., Jeong M. H., Kim J.-S. et al. (2011). Clinical benefit of statin pretreatment in patients undergoing percutaneous coronary intervention: a collaborative patient-level meta-analysis of 13 randomized studies. *Circulation* 123, 1622-1632. 10.1161/CIRCULATIONAHA.110.002451 21464051

[DMM018713C35] Pearse R. M., Moreno R. P., Bauer P., Pelosi P., Metnitz P., Spies C., Vallet B., Vincent J.-L., Hoeft A. and Rhodes A. (2012) Mortality after surgery in Europe: a 7 day cohort study. *Lancet* 380, 1059-1065. 10.1016/S0140-6736(12)61148-9 22998715 PMC3493988

[DMM018713C36] Plump A. S., Smith J. D., Hayek T., Aalto-Setälä K., Walsh A., Verstuyft J. G., Rubin E. M. and Breslow J. L. (1992). Severe hypercholesterolemia and atherosclerosis in apolipoprotein E-deficient mice created by homologous recombination in ES cells. *Cell* 71, 343-353. 10.1016/0092-8674(92)90362-G 1423598

[DMM018713C37] Potteaux S., Gautier E. L., Hutchison S. B., van Rooijen N., Rader D. J., Thomas M. J., Sorci-Thomas M. G. and Randolph G. J. (2011). Suppressed monocyte recruitment drives macrophage removal from atherosclerotic plaques of Apoe−/− mice during disease regression. *J. Clin. Invest.* 121, 2025-2036. 10.1172/JCI43802 21505265 PMC3083793

[DMM018713C38] Priebe H.-J. (2011). Preoperative cardiac management of the patient for non-cardiac surgery: an individualized and evidence-based approach. *Br. J. Anaesth.* 107, 83-96. 10.1093/bja/aer121 21610016

[DMM018713C39] Rattazzi M., Bennett B. J., Bea F., Kirk E. A., Ricks J. L., Speer M., Schwartz S. M., Giachelli C. M. and Rosenfeld M. E. (2005). Calcification of advanced atherosclerotic lesions in the innominate arteries of ApoE-deficient mice: potential role of chondrocyte-like cells. *Arterioscler. Thromb. Vasc. Biol.* 25, 1420-1425. 10.1161/01.ATV.0000166600.58468.1b 15845913

[DMM018713C40] Ross R. (1999). Atherosclerosis--an inflammatory disease. *N. Engl. J. Med.* 340, 115-126. 10.1056/NEJM199901143400207 9887164

[DMM018713C41] Sasaki T., Kuzuya M., Nakamura K., Cheng X. W., Shibata T., Sato K. and Iguchi A. (2006). A simple method of plaque rupture induction in apolipoprotein E-deficient mice. *Arterioscler. Thromb. Vasc. Biol.* 26, 1304-1309. 10.1161/01.ATV.0000219687.71607.f7 16574894

[DMM018713C42] Schieffer B., Selle T., Hilfiker A., Hilfiker-Kleiner D., Grote K., Tietge U. J. F., Trautwein C., Luchtefeld M., Schmittkamp C., Heeneman S. et al. (2004). Impact of interleukin-6 on plaque development and morphology in experimental atherosclerosis. *Circulation* 110, 3493-3500. 10.1161/01.CIR.0000148135.08582.97 15557373

[DMM018713C43] Schuett H., Oestreich R., Waetzig G. H., Annema W., Luchtefeld M., Hillmer A., Bavendiek U., von Felden J., Divchev D., Kempf T. et al. (2012). Transsignaling of interleukin-6 crucially contributes to atherosclerosis in mice. *Arterioscler. Thromb. Vasc. Biol.* 32, 281-290. 10.1161/ATVBAHA.111.229435 22075248

[DMM018713C44] Shah P. K., Yano J., Reyes O., Chyu K.-Y., Kaul S., Bisgaier C. L., Drake S. and Cercek B. (2001). High-dose recombinant apolipoprotein A-I(milano) mobilizes tissue cholesterol and rapidly reduces plaque lipid and macrophage content in apolipoprotein e-deficient mice: potential implications for acute plaque stabilization. *Circulation* 103, 3047-3050. 10.1161/hc2501.092494 11425766

[DMM018713C45] Stary H. C. (2000). Natural history and histological classification of atherosclerotic lesions: an update. *Arterioscler. Thromb. Vasc. Biol.* 20, 1177-1178. 10.1161/01.ATV.20.5.1177 10807728

[DMM018713C46] Theilmeier G., De Geest B., Van Veldhoven P. P., Stengel D., Michiels C., Lox M., Landeloos M., Chapman M. J., Ninio E., Collen D. et al. (2000). HDL-associated PAF-AH reduces endothelial adhesiveness in apoE−/− mice. *FASEB J.* 14, 2032-2039. 10.1096/fj.99-1029com 11023987

[DMM018713C47] Tietge U. J. F., Maugeais C., Cain W., Grass D., Glick J. M., de Beer F. C. and Rader D. J. (2000). Overexpression of secretory phospholipase A(2) causes rapid catabolism and altered tissue uptake of high density lipoprotein cholesteryl ester and apolipoprotein A-I. *J. Biol. Chem.* 275, 10077-10084. 10.1074/jbc.275.14.10077 10744687

[DMM018713C48] van der Meij E., Koning G. G., Vriens P. W., Peeters M. F., Meijer C. A., Kortekaas K. E., Dalman R. L., van Bockel J. H., Hanemaaijer R., Kooistra T. et al. (2013). A clinical evaluation of statin pleiotropy: statins selectively and dose-dependently reduce vascular inflammation. *PLoS ONE* 8, e53882 10.1371/journal.pone.0053882 23349755 PMC3551939

[DMM018713C49] Werner N., Nickenig G. and Laufs U. (2002). Pleiotropic effects of HMG-CoA reductase inhibitors. *Basic Res. Cardiol.* 97, 105-116. 10.1007/s003950200000 12002257

[DMM018713C50] Willerson J. T. and Ridker P. M. (2004). Inflammation as a cardiovascular risk factor. *Circulation* 109, II2-I10. 10.1161/01.CIR.0000079427.64438.8f 15173056

[DMM018713C51] Winterhalter M., Brandl K., Rahe-Meyer N., Osthaus A., Hecker H., Hagl C., Adams H. A. and Piepenbrock S. (2008). Endocrine stress response and inflammatory activation during CABG surgery. A randomized trial comparing remifentanil infusion to intermittent fentanyl. *Eur. J. Anaesthesiol.* 25, 326-335. 10.1017/S0265021507003043 18005471

[DMM018713C52] Wu M. S., Robbins J. C., Bugianesi R. L., Ponpipom M. M. and Shen T. Y. (1981). Modified in vivo behavior of liposomes containing synthetic glycolipids. *Biochim. Biophys. Acta* 674, 19-29. 10.1016/0304-4165(81)90342-1 7236728

